# Gene model for the ortholog of *PGRP-SB1* in *Drosophila funebris*

**DOI:** 10.17912/micropub.biology.002270

**Published:** 2026-07-07

**Authors:** Molly McDonald, Pablo Chialvo, Clare Scott Chialvo

**Affiliations:** 1 Biology, Appalachian State University, Boone, North Carolina USA

## Abstract

We developed a gene model for the *Peptidoglycan recognition protein SB1* ortholog ( *PGRP-SB1* ) in the ASM1890182v1 Genome Assembly (GenBank Accession: GCA_018901825.1 ) of *Drosophila funebris* . This ortholog was characterized as part of a developing dataset for a comparative study of detoxification gene family evolution in the *immigrans* - *tripunctata* radiation of the genus *Drosophila* using an adapted Genomics Education Partnership gene annotation protocol for Course-based Undergraduate Research Experiences.

**Figure 1. Genomic neighborhood and gene model for PGRP-SB1 in D. funebris:** (A) **Synteny comparison of the genomic neighborhoods for *PGRP-SB1* in *Drosophila melanogaster* and *Drosophila funebris* .** Thin underlying arrows indicate which DNA strand the target gene– *PGRP-SB1* –is located on in *D. melanogaster* (top) and *D. funebris* (bottom). The thin arrows pointing to the left indicate that *PGRP-SB1* is on the negative strand in both *D. melanogaster* and *D. funebris* . The wide gene arrows pointing in the same direction as *PGRP-SB1* are on the same strand relative to the thin underlying arrows, while wide gene arrows pointing in the opposite direction of *PGRP-SB1* are on the opposite strand relative to the thin underlying arrows. White gene arrows in *D. funebris* indicate orthology to the corresponding gene in *D. melanogaster* , while black gene arrows indicate non-orthology and grey gene arrows indicate that the gene is present in both neighborhoods but not syntenic. Gene symbols given in the *D. funebris* gene arrows indicate the orthologous gene in *D. melanogaster* , while the locus identifiers are specific to *D. funebris* . (B) **Gene Model in GEP UCSC Track Data Hub** (Raney et al., 2014). The coding-regions of *PGRP-SB1* in *D. funebris* are displayed in the User Supplied Track (red); coding sequences (CDS) are depicted by thick rectangles and introns by thin lines with arrows indicating the direction of transcription. Subsequent evidence tracks include Spaln of *D. melanogaster* Proteins (purple, alignment of Ref-Seq proteins from *D. melanogaster* ), Coding Regions Predicted by Augustus (dark blue), GeMoMa (teal), and NSCAN PASA-EST (dark green), and RNA-Seq from mixed sex adult flies (brown; alignment of Illumina RNA-Seq reads from *D. funebris* – Erlenbach et al., 2023). (C) **Dot Plot of PGRP-SB1-PA in *D. melanogaster* ( *x* -axis) vs. the orthologous peptide in *D. funebris* ( *y* -axis).** Amino acid number is indicated along the left and bottom; CDS number is indicated along the top and right, and CDSs are also highlighted with alternating colors. Line breaks in the dot plot indicate areas of with low sequence identity between species. One longer break is present that extends across all of CDS 1 and the beginning of CDS 2 (dark purple box – a). A smaller break is also present towards the end of CDS 2 (yellow box – b). (D) **Idiosyncrasies in protein alignment.** The protein model contains one longer break and a shorter break that indicate areas of low sequence identity. In the longer break that extends across all of CDS 1 and into CDS 2 (dark purple box – a), only two of the 25 amino acids are highly dissimilar. There is one fewer amino acid in CDS 1 of *D. funebris* , and an insertion of three amino acids is present at the beginning of CDS 2 of *D. funebris* . The shorter break that occurs towards the end of CDS 2 (yellow box – b) spans a region of 11 amino acids, only one of which is highly dissimilar.

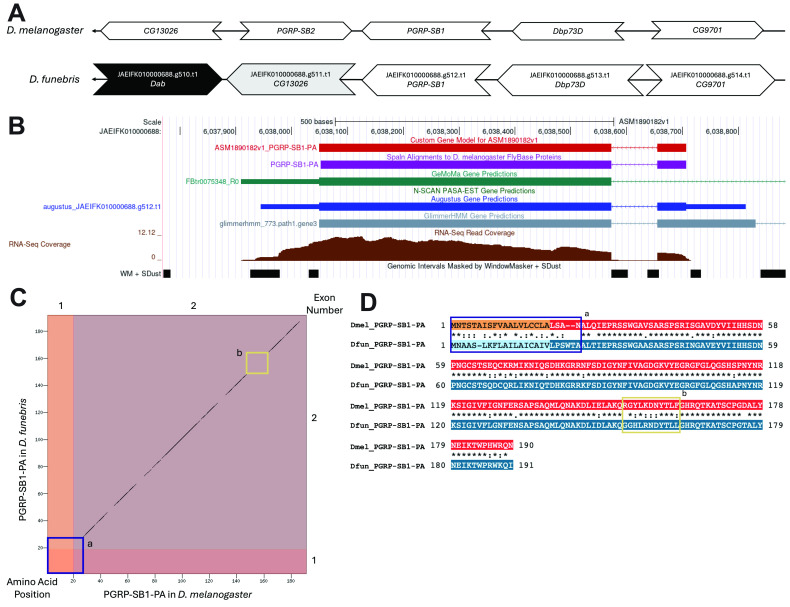



## Description

**Table:** 

* This article reports a predicted gene model generated by undergraduate work using a structured gene model annotation protocol defined by the Genomics Education Partnership (GEP; thegep.org ) for Course-based Undergraduate Research Experience (CURE). The following information in quotes may be repeated in other articles submitted by participants using the same GEP CURE protocol for annotating Drosophila species orthologs of Drosophila melanogaster detoxification genes. * “Within insects, the process of detoxifying xenobiotics and host secondary metabolites is a three-phase process that involves functionalization, conjugation, and excretion of these compounds. Expansions of known detoxification gene families ( *e.g.* , cytochrome P450s) is associated with diet breadth and insecticide resistance (Ranson et al., 2002; Després et al., 2007; Rane et al., 2016). With the increasing availability of high-quality genomes for non-model organisms, including *Drosophila* species beyond *D. melanogaster* , it is now possible to perform large scale comparative studies (Robinson et al., 2011; Kim et al., 2021; Threfall and Baxter, 2021). Careful manual annotation and curation of gene models can improve upon computational gene predictions in non-model species, which aids the accuracy of studies on gene and genome evolution (Mudge and Harrow, 2016; Tello-Ruiz et al., 2019). To aid in these annotations, the Genomics Education Partnership (thegep.org) developed a curriculum involving web-based tools that allow undergraduates to engage in authentic course-based research focused on manually annotating genes in non-model species (Rele et al., 2023). The orthologous gene models, including the one presented here, then provide a reliable basis for further evolutionary genomic analyses when made available to the scientific community. The gene ortholog described here in *D. funebris* for *Peptidoglycan recognition protein SB1* ( *PGRP-SB1* ), a member of the peptidoglycan recognition protein family that contributes to the innate immune system of insects, was characterized as part of a developing dataset for a comparative study of detoxification gene families in the *immigrans* - *tripunctata* radiation of the genus *Drosophila* .” (Williams et al., 2026) *Drosophila funebris* (Fabricius, 1787) is a member of the *funebris* species group, which occurs in the *immigrans-tripunctata* radiation of the *Drosophila* subgenus (Bächli, 2005; ICZN, 2010). It is also the type species of the genus *Drosophila* . This species is a globally distributed human commensal (Grimaldi, 2022). While *D. funebris* feeds on shelf fungi and fruit (Kimura et al., 1977; Prigent et al., 2003), it does not tolerate the mushroom toxin α-amanitin (Stump et al., 2011; Erlenbach et al., 2023).

When considering the detoxification of xenobiotics, the focus is often directed towards genes that produce detoxification enzymes. A growing body of work is beginning to highlight the role of genes in the insect's innate immune system in metabolizing these toxic compounds (Bartling et al., 2021; Jiang et al., 2021; Rahmat et al., 2024). One component of the innate immune system, peptidoglycan recognition proteins (PGRP), are pattern recognition molecules that detect molecular patterns found in pathogens but absent in the host organism (Akira et al., 2006; Lemaitre and Hoffman, 2007). Within *Drosophila melanogaster* , Werner et al. (2000) identified 12 PGRPs. These proteins are split into two subgroups (long and short) based on their transcript size, cellular location, and function (Werner et al., 2000). *Peptidoglycan recognition protein SB1* ( *PGRP-SB1 )* is a short PGRP (Werner et al., 2000). It is an amidase that exhibits antibacterial activity against peptidoglycans that possess a diaminopimelic acid residue (Mellroth and Steiner, 2006). When *Drosophila* flies are exposed to wasp, mite, or nematode parasites, the expression of this gene is increased (Benoit et al., 2020). In addition, exposure to sublethal doses of six insecticides also increased the expression of *PGRP-SB1* (Gao et al., 2021).

We propose a gene model for the *D. funebris* ortholog of the *D. melanogaster* *Peptidoglycan recognition protein SB1* ( *PGRP-SB1* ) gene. The genomic region of the ortholog corresponds to the Augustus gene prediction JAEIFK010000688 .g512.t1 in the ASM1890182v1 Genome Assembly of *D. funebris* (GCA_018901825.1- Kim et al. 2021). This model is based on mixed sex, adult RNA-Seq data from *D. funebris* (Erlenbach et al. 2023; https://doi.org/10.5061/dryad.hdr7sqvq2 ) and *PGRP-SB1* in *D. melanogaster* using FlyBase release FB2024_02 ( GCA_000001215.4 ; Gramates et al., 2022; Jenkins et al., 2022; Larkin et al., 2021).

***Synteny***

The reference gene, *PGRP-SB1 ,* occurs on chromosome 3L in *D. melanogaster* and is flanked upstream by *Dead box protein 73D* ( *Dbp73D* ) and *CG9701* and downstream by *Peptidoglycan recognition protein SB2* ( *PGRP-SB2* ) and *CG13026* . The *tblastn* search of *D. melanogaster* PGRP-SB1-PA (query) against the *D. funebris* (GenBank Accession: GCA_018901825.1 Genome Assembly database (subject) placed the putative ortholog of *PGRP-SB1* within contig_693 ( JAEIFK010000688 ) which corresponds to the Augustus gene prediction JAEIFK010000688 .g512.t1 (E-value: 3.00e-113; percent identity: 78.2%; query coverage: 98% as determined by *blastp* ). The putative ortholog is flanked upstream by Augustus gene predictions JAEIFK010000688 .g513.t1 and JAEIFK010000688 .g514.t1, which correspond to *Dbp73D* and *CG9701* in *D. melanogaster* (E-value: 0.0 and 0.0; identity: 66.47% and 88.70%, respectively, as determined by *blastp* ; Figure 1A ; Altschul et al., 1990). The putative ortholog of *PGRP-SB1* is flanked downstream by JAEIFK010000688 .g511.t1 and JAEIFK010000688 .g510.t1, which correspond to *CG13026* and *Disabled* ( *Dab* ) in *D. melanogaster* (E-value: 1.00e-13 and 0.0 identity: 52.75% and 68.16%, respectively, as determined by *blastp* ). *Dab* is the third gene downstream of the target gene, *PGRP-SB1* , in *D. melanogaster* . The putative ortholog assignment for *PGRP-SB1* in *D. funebris* is supported by the following evidence: The gene predictions surrounding the *PGRP-SB1* ortholog are all orthologous to the genes at the same locus in *D. melanogaster* except for the loss of the *PGRP-SB2* gene, gene expression data corresponds with each prediction, and local synteny is almost entirely conserved, supported by E-values and percent identities, so we conclude that JAEIFK010000688 .g512.t1 is an ortholog of *PGRP-SB1* in *D. funebris* ( Figure 1A ).

***Protein Model***

*PGRP-SB1* in *D. funebris* has 2 coding sequences (CDS) within the genome sequence. The only unique protein sequence (PGRP-SB1-PA) is translated from 1 mRNA isoform (PGRP-SB1-RA; Figure 1B ). Relative to the ortholog in *D. melanogaster* , the CDS number and protein isoform count are conserved *.* The sequence of PGRP-SB1-PA in *D. funebris* has 78.1% identity (89.1% similarity) with the protein-coding isoform PGRP-SB1-PA in *D. melanogaster* , as determined by *blastp* ( Figure 1C ). This level of divergence is not surprising given that *D. funebris* and *D. melanogaster* belong to two separate subgenera ( *Drosophila* and *Sophophora* respectively) that diverged approximately 45-60 MYA (Russo et al., 1995; Tamura et al., 2004; Obbard et al., 2012). Coordinates of this curated gene model are archived in the CaltechDATA repository (see “Extended Data” section below).

## Methods

The annotation methods used in this project are adapted from those described in Rele et al. (2023), which includes algorithms, database versions, and citations for the complete annotation process developed for the Pathways Project. The methods for the current project are detailed in brief below with notes on significant differences between this protocol and the one described in Rele et al. (2023). The students use the GEP instance of the UCSC Genome Browser v.435 ( https://gander.wustl.edu ; Kent et al., 2002; Raney et al., 2024) to examine the genomic neighborhood of their reference detoxification gene in the *D. melanogaster* genome assembly (Aug. 2014; BDGP Release 6 + ISO1 MT/dm6). Students obtain the protein sequence for the *D. melanogaster* target gene for a given isoform and use a *tblastn* search of the sequence against their target *Drosophila* species genome assembly ( *D. funebris* ( GCA_018901825.1 – Kim et al., 2013)) on the NCBI BLAST server ( https://blast.ncbi.nlm.nih.gov/Blast.cgi , Altschul et al., 1990) to identify the putative ortholog location. Students compare the genomic neighborhood of the putative ortholog to that of the reference gene in *D. melanogaster* . This local synteny analysis includes a minimum of two upstream and downstream genes relative to the potential ortholog. As no RefSeq protein data is available for these species, comparisons are based on gene predictions that correlate with gene expression data in the putative ortholog neighborhood. Using the multiple alignment tracks feature in the Genome Browser, students examine other sets of genomic evidence, including Spaln alignment of *D. melanogaster* proteins, multiple gene prediction tracks (e.g., GeMoMa, Augustus, NSCAN PASA-EST), and mixed sex RNA-Seq adult expression data from the target species generated by Erlenbach et al. (2023; https://doi.org/10.5061/dryad.hdr7sqvq2 ). Information on the genomic structure information (e.g., CDSs, intron-exon number, number of isoforms) for the reference gene in *D. melanogaster* is retrieved using Gene Record Finder ( https://gander.wustl.edu/~wilson/dmelgenerecord/index.html ; Rele et al *.,* 2023). To determine approximate splice sites within the target gene, a *tblastn* search using the CDSs from the *D. melanogaste* r reference gene against the putative ortholog location (10kb up- and downstream of the target gene prediction). Coordinates of the CDS(s) are refined by examining aligned RNA-Seq data, identifying canonical splice site sequences, and ensuring the maintenance of an open reading frame. Students confirm the biological validity of their target gene model using the FlySeq Gene Model Checker ( https://gander2.wustl.edu/~wilson/genechecker-flyseq/ ), which compares the hypothesized target gene model's structure and translated sequence against the *D. melanogaster* reference gene. At least two independent models for this gene are generated. These models are reconciled by a third independent researcher to produce the final model presented here. Note: comparison of 5' and 3' UTR sequence information is not included in this GEP CURE protocol.

## Data Availability

Description: Zipped archive containing FASTA, PEP, and GFF. Resource Type: Dataset. DOI: https://doi.org/10.22002/w8r9v-73q82
